# Interatrial block in prediction of all-cause mortality after first-ever ischemic stroke

**DOI:** 10.1186/s12872-019-1015-5

**Published:** 2019-02-11

**Authors:** M. A. Baturova, A. Lindgren, Y. V. Shubik, J. Carlson, P. G. Platonov

**Affiliations:** 1 Department of Cardiology, Clinical Sciences, Lund University, SE-221 85 Lund, Sweden; 2 Research Park, St Petersburg State University, Peterhof, Botanicheskaya, 17 St Petersburg, Russia; 3 Department of Neurology and Rehabilitation Medicine, Skåne University Hospital, Lund, Sweden; 4 Cardiology research, clinical and educational center, St. Petersburg State University, Universitetskaya Embankment, 7/9, St. Petersburg, Russia; 5 Arrythmia Clinic, Skåne University Hospital, SE-221 85 Lund, Sweden

**Keywords:** Interatrial block, All-cause mortality, Ischemic stroke, ECG, Atrial fibrosis

## Abstract

**Background:**

Interatrial block (IAB) is an ECG indicator of atrial fibrosis related to atrial remodeling and thrombus formation thus leading to embolic stroke and increasing mortality. We aimed to assess weather IAB predicted all-cause mortality during 10 years after ischemic stroke.

**Methods:**

The study sample comprised 235 patients (median age 74 (interquartile range 25–75% 65–81) years, 95 female) included in the Lund Stroke Register in 2001–2002, who had sinus rhythm ECGs at stroke admission. IAB was defined as a P-wave duration ≥120 ms without = partial IAB (*n* = 56) or with = advanced IAB (*n* = 41) biphasic morphology (±) in the inferior ECG leads. All-cause mortality was assessed via linkage with the Swedish Causes of Death Register.

**Results:**

During follow-up 126 patients died (54%). Advanced IAB, but not partial, was associated with all-cause mortality in univariate Cox regression analysis (hazard ratio (HR) 1.98, 95% CI 1.27–3.09, *p* = 0.003). After adjustment for age, gender, severity of stroke measured by NIHSS scale and smoking status in patients without additional comorbidities advanced IAB independently predicted all-cause mortality (HR 7.89, 95% CI 2.01–30.98, *p* = 0.003), while in patients with comorbidities it did not (HR 1.01 95% CI 0.59–1.72, *p* = 0.966).

**Conclusion:**

Advanced IAB predicted all-cause mortality after ischemic stroke, but mostly in patients without additional cardiovascular comorbidities.

## Background

Interatrial block (IAB) was first described as an electrocardiographic (ECG) phenomenon reflecting delayed conduction between the right and left atria during sinus rhythm in 1980s by Bayés de Luna [1]. IAB is defined as P-wave duration exceeding 120 ms. In case of biphasic (+/−) P wave in inferior leads it was considered to be advanced IAB [2].

Though the association between IAB and atrial fibrillation (AF) has been well established [1, 3], the prognostic value of IAB in prediction of all-cause mortality is insufficiently documented. IAB has been linked to underlying atrial fibrosis occurring as a result of ageing or cardiovascular comorbidities [4] and is related to atrial remodeling, AF and thromboembolism, factors that are associated with increased mortality. Recently, it has been shown that IAB was associated with cardiovascular and all-cause mortality in a general population [5] and predicted cardioembolic stroke [6]. However, to the best of our knowledge the literature data on mortality in the context of IAB pattern in high-risk populations are scarce.

We aimed to assess the association of IAB with all-cause mortality in first-ever ischemic stroke survivors during 10 years after stroke.

## Methods

### Study cohort

The original study population comprised of 336 consecutive first-ever ischemic stroke patients included in the Lund Stroke Register (LSR) between March 1, 2001 and February 28, 2002, during the first year of the LSR existence [7]. Informed consent was obtained from all participants included in the LSR. The Lund University Ethics Committee approved the study.

As the main objective of the study was related to the presence of IAB at admission with ischemic stroke, a requirement was availability of ECGs with presence of P waves suitable for this analysis. The inclusion of patients for this study was therefore based on the accessibility of sinus rhythm ECGs exportable in a digital format at stroke admission. After exclusion patients with AF on baseline ECG (*n* = 60, 44 with permanent AF and 16 with non-permanent AF as described previously [7]) and patients with either non-sinus rhythm at admission or insufficient ECG quality, 243 patients remained. We did not exclude patients with a history of non-permanent AF presenting with sinus rhythm at stroke onset if they had available sinus rhythm ECG.

During the hospital stay 8 patients died and could not enter the prospective follow-up. The sample size therefore was limited to 235 subjects (median age 74 years (IQR 65–81), 95 female) with sinus rhythm on ECG at inclusion in the LSR at stroke onset who were discharged alive after their first-ever ischemic stroke (Table 1).

**Table 1:** Baseline characteristics of the patients and the number of died during follow-up

Variable	All patients *n* = 235	No IAB *n* = 138	Partial IAB *n* = 56	Advanced IAB *n* = 41	*P* value	Intermediate risk patients *n* = 47	High-risk patients *n* = 188	*P* value	Patients without AF *n* = 186
	1	2	3	4	2–3-4	5	6	5–6	7
Age, years, median (IQR)	74 (65–81)	71 (61–80)	76 (69–82)	79 (74–86)	0.001	74 (57–80)	74 (67–81)	0.229	74 (62–80)
Female gender, n (%)	95 (40)	52 (38)	27 (48)	16 (39)	0.391	21 (45)	74 (39)	0.511	75 (40)
Diabetes, n (%)	38 (16)	28 (17)	7 (13)	6 (15)	0.603	0	38 (20)	< 0.001	27 (15)
Vascular diseases, n (%)	101 (43)	54 (39)	29 (52)	18 (44)	0.270	0	101 (54)	< 0.001	80 (43)
Hypertension, n (%)	139 (59)	79 (57)	38 (68)	22 (53)	0.290	0	139 (74)	< 0.001	107 (58)
Cardiac failure, n (%)	14 (6)	9 (7)	3 (5)	2 (5)	0.905	0	14 (7)	0.341	7 (4)
History of non-permanent AF before stroke onset, n (%)	49 (21)	27 (20)	15 (27)	7 (17)	0.430	0	49 (26)	< 0.001	NA
NIHS scale, median (IQR)	3 (2–6)	3 (2–6)	4 (2–11)	4 (2–8)	0.161	3 (1–5)	3 (2–7)	0.173	3 (2–6)
Oral anticoagulants, n (%)	43 (18)	26 (19)	14 (25)	3 (7)	0.098	3 (6)	40 (21)	0.019	22 (12)
Advanced IAB, n (%)	41 (17)	0	0	NA	NA	11 (23)	30 (16)	0.281	34 (18)
Died during follow-up, n (%)	126 (54)	64 (46)	32 (57)	30 (73)	0.009	22 (47)	104 (55)	0.329	96 (52)

### Baseline clinical and ECG assessment

Medical records of all study subjects were analyzed for the baseline clinical characteristics. Stroke severity was estimated using the National Institutes of Health Stroke Scale (NIHSS) [8].

**Fig. 1:**
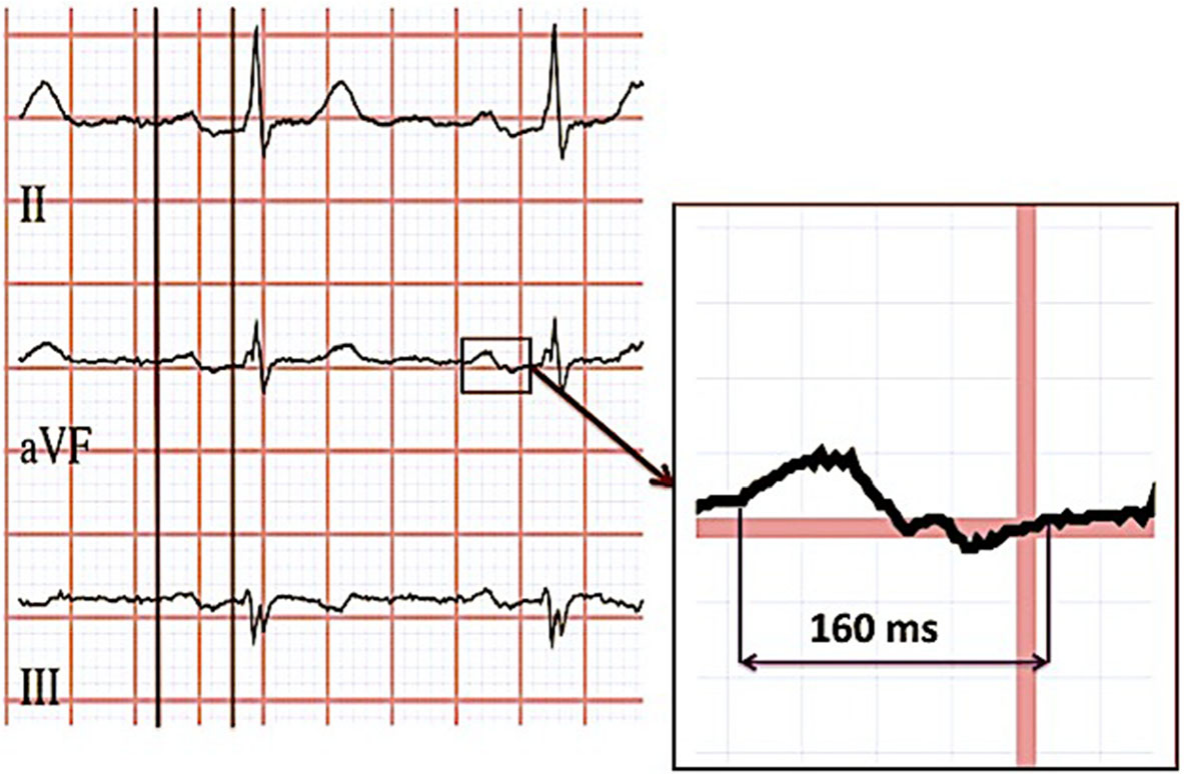
ECG example of prolonged P wave duration with biphasic (+/−) configuration in inferior leads – advanced interatrial block

Sinus rhythm ECGs were extracted from the regional electronic database (GE MUSE, GE Healthcare, MegaCare) and processed offline. The assessment of P-wave duration, measured in milliseconds (ms), and P-wave configuration (positive or biphasic) was performed automatically using the University of Glasgow 12-lead ECG analysis algorithm [9]. IAB was defined according to the diagnostic criteria with a P-wave ≥120 ms [2]. Partial IAB was defined as a P-wave ≥120 ms but with no negative terminal part detected in the inferior leads II, III or aVF. Advanced IAB was considered to be present when a P-wave ≥120 ms and a negative terminal part was detected in one of the inferior leads II, III or aVF, Fig. 1.

### Ascertainment of study endpoints and the use of anticoagulant therapy

We followed up all study subjects until October 17, 2011. The median duration of follow-up was 9 (IQR 4–10) years. Dates of death, primary and secondary diagnoses on the date of death were determined via record linkage with the Swedish Causes of Death Register as reported previously [10]. The endpoint in this study was defined as all-cause mortality.

Oral anticoagulant (OAC) therapy at discharge and during the 10-year follow-up was ascertained using the Lund University Hospital anticoagulation database, which contains data for all patients in the hospital’s local catchment area receiving OAC [10]. Non-vitamin K antagonist oral anticoagulants were not available at the time of enrollment in the LSR, so in our study OAC therapy was limited to the use of warfarin.

### Statistical methods

Baseline clinical characteristics were compared between patients without IAB and with partial IAB or advanced IAB using chi-square or Fisher’s exact test for categorical variables. Non-parametric tests were used for continuous variables. Binary logistic regression analysis was performed to evaluate odds ratios (OR) and 95% confidence interval (CI) of the association between the baseline clinical characteristics and the presence of partial and advanced IAB.

All-cause mortality was analyzed with regard to the presence and the degree of IAB (partial or advanced). The Kaplan-Meier product-limit method was used to generate a survival curve indicating survival during the 10-year follow-up after the first-ever ischemic stroke. Patients who remained alive were censored at the end of the follow-up period. Differences between groups were tested using the log-rank test.

Cox proportional hazard regression models were used to estimate the hazard ratios (HR) and their 95% CI of all-cause mortality associated with partial IAB or advanced IAB. Hazards proportional assumption was checked using graphical method generating log minus log plot. In multivariate Cox regression analyses we included age, gender, cardiac failure, hypertension, non-permanent AF, vascular disease, diabetes, severity of stroke measured by NIHSS scale and smoking status, the factors known to be associated with all-cause mortality in stroke survivors [10, 11].

Since P-wave duration has been reported to predict increased mortality rate in patients without cardiovascular comorbidities [5], we performed Kaplan Meier curve and Cox regression analyses separately for stroke survivors without additional cardiovascular comorbidities (these subjects were considered to be intermediate-risk patients) as well as for the group of patients with one or more additional cardiovascular comorbidities such as hypertension, ischemic heart diseases, cardiac failure, non-permanent AF or diabetes (defined as high-risk patients). Due to the small number of partial IAB cases (*n* = 4) in patients with intermediate cardiovascular risk among whom only one patient died, we performed Kaplan-Meier curve analysis comparing patients with advanced IAB and those who did not have advanced IAB. For the sake of comparability the same grouping was performed in the group of patients with high cardiovascular risk.

Also we evaluated if mortality was associated with the use of OAC therapy in relation to the presence of IAB at baseline. For that purpose we excluded subjects with established AF diagnosis at discharge from index admission with ischemic stroke from analysis (*n* = 49). Among the remaining 186 patients (median age 74, IQR 62–80 years, 75 females) only 2 patients treated with OAC had advanced IAB. Therefore, patients with either partial IAB or advanced IAB (*n* = 75 of 186 subjects) were categorized as having IAB and were compared with patients with normal P-wave duration at baseline (*n* = 111 of 186 subjects). Kaplan Meier analysis was performed to estimate the impact of OAC on survival in patients with and without IAB.

*P*-values of < 0.025 were considered significant. All analyses were performed using SPSS Statistics 25 (SPSS Inc., Chicago, Illinois, USA).

## Results

### Patient characteristics and oral anticoagulant therapy at baseline

Partial IAB was observed in 56 (24%) and advanced IAB - in 41 (17%) of the 235 patients. Baseline clinical characteristics are presented in the Table 1.

Patients with either partial IAB or advanced IAB were older than those without IAB, but did not differ regarding other clinical factors and the use of OAC after ischemic stroke, which generally was low (*n* = 43). In univariate logistic regression analysis only age was significantly associated with partial and advanced IAB (Table 2).

**Table 2:** Univariate binary logistic regression analysis showing the association between baseline clinical characteristics and presence of interatrial block (IAB)

Covariate	Presence of partial IAB			Presence of advanced IAB		
	OR	95% CI	P value	OR	95% CI	P value
Age at stroke admission	1.05	1.02–1.09	0.001	1.07	1.03–1.11	0.001
Age more 65 years	5.62	1.93–16.31	0.002	3.57	1.21–10.49	0.021
Age more 75 years	1.17	0.87–1.58	0.311	1.93	1.33–2.80	0.001
Diabetes	0.65	0.26–1.59	0.342	0.87	0.34–2.23	0.769
Vascular diseases	1.67	0.89–3.12	0.108	1.05	0.53–1.06	0.895
Hypertension	1.58	0.82–3.03	0.173	0.76	0.39–1.75	0.432
Cardiac failure	0.81	0.21–3.12	0.761	0.78	0.17–3.62	0.749
Non-permanent AF	1.50	0.73–3.11	0.270	0.75	0.31–1.80	0.513

At enrolment, 47 patients (20%) comprised a group of stroke patients with intermediate cardiovascular risk i.e. without hypertension, ischemic heart diseases, cardiac failure, non-permanent AF or diabetes. They did not differ in regard to age, gender or the prevalence of advanced IAB from the high-risk patients (*n* = 188, 80%), Table 1.

At discharge after the index stroke admission 29 patients were receiving warfarin: 17 of them due to AF, 6 – due to venous thrombosis and 6 – due to undocumented cause. Within 0.5 (IQR 0.3–4) year from the stroke onset additional 14 patients received treatment: 4 of them due to AF known at baseline, 7 of them due to new onset AF and 3 of them due to venous thrombosis. Thus in total, 43 patients (18% of the whole study population) were treated with OAC during follow-up, among whom 21 patients had non-permanent AF documented by baseline (43% of all patients with documented non-permanent AF). Median duration of OAC therapy was 8 (IQR 1–9) years.

### All-cause mortality

**Fig. 2:**
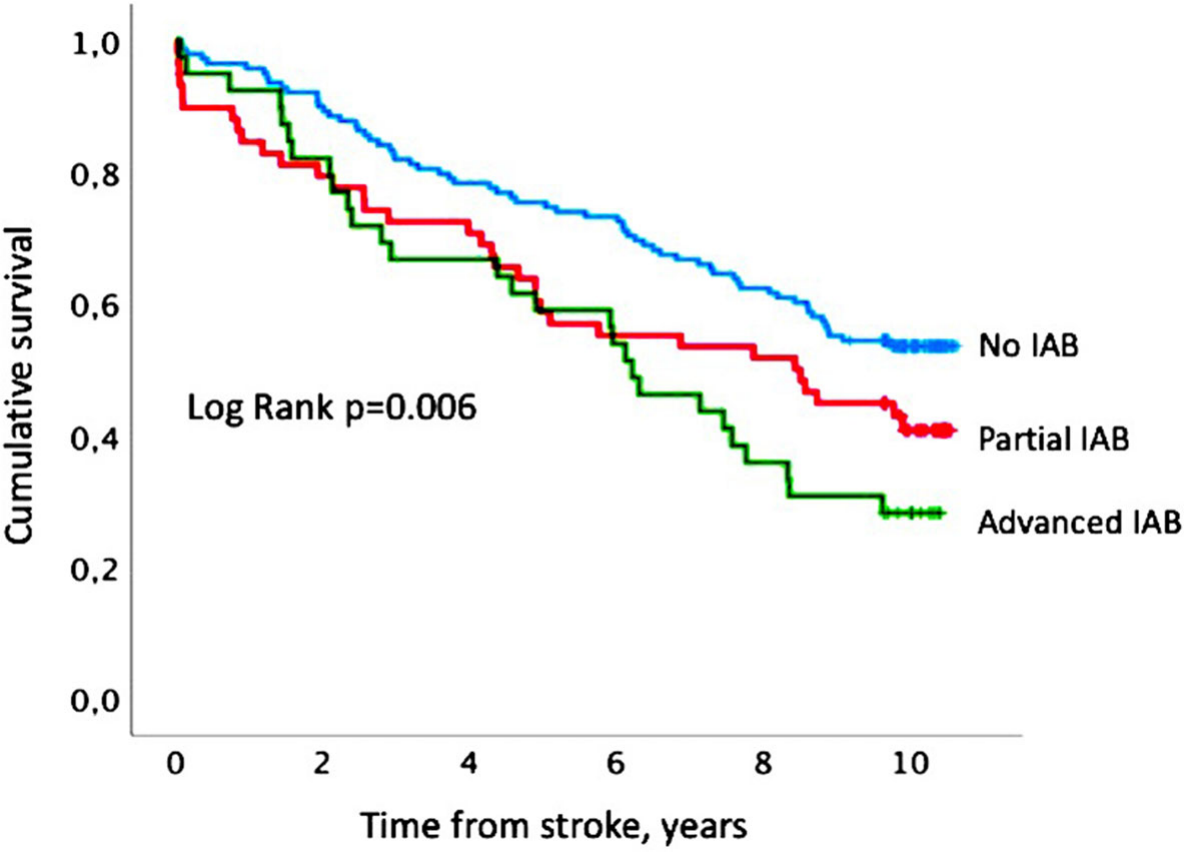
Kaplan-Meier survival curve indicating survival in accordance to the degree of ineratrial block (IAB) in stroke patients

During follow-up 126 patients died (54%): 64 patients with normal P-wave duration (46%), 32 patients with partial IAB (57%) and 30 patients with advanced IAB (73%), *p* = 0.009, Table 1. Kaplan-Meier survival curves categorized by the presence and the degree of IAB are presented in Fig. 2. The total mortality was similar among patients with intermediate (*n* = 22, 47%) and high cardiovascular risk (*n* = 104, 55%; *p* = 0.329). The vast majority of patients who died during follow-up in both groups were among subjects with advanced IAB (Table 3).

**Table 3:** Death rate during follow-up within groups of patients with intermediate cardiovascular risk and high cardiovascular risk in regard to the presence and degree of IAB

Group	Total	Advanced IAB	Partial IAB	No IAB
Intermediate cardiovascular risk				
Number of patients, n (%)	47 (100)	11 (23)	4 (8)	32 (68)
Died during follow-up, n (%)	22 (47)	8 (73)	1 (25)	13 (41)
High cardiovascular risk				
Number of patients, n (%)	188 (100)	30 (16)	52 (28)	106 (56)
Died during follow-up, n (%)	104 (55)	22 (73)	31 (60)	51 (48)

*IAB* interatrial block on ECG at stroke onset

In the univariate Cox regression analysis, advanced IAB, but not partial IAB, was significantly associated with increased risk of death (Table 4). However, in the multivariate Cox regression analysis advanced IAB was no longer a significant predictor of death.

**Table 4:** Cox regression analysis showing the value of interatrial block (IAB) for prediction of all-cause mortality after first-ever ischemic stroke

Variable	Univariate analysis			Multivariate analysis		
	HR	95% CI	P value	HR	95% CI	P value
Partial IAB vs no IAB	1.48	0.98–2.24	0.065	1.06	0.68–1.65	0.808
Advanced IAB vs no IAB	1.98	1.27–3.09	0.003	1.14	0.72–1.82	0.568
Age	1.08	1.06–1.10	< 0.001	1.09	1.07–1.12	< 0.001
Male gender	1.03	0.72–1.47	0.887	1.66	1.12–2.47	0.012
Cardiac failure	2.30	1.23–4.28	0.009	134	0.68–2.67	0.401
Diabetes	1.11	0.70–1.78	0.654	0.82	0.49–1.37	0.451
Hypertension	1.28	0.89–1.83	0.190	1.22	0.83–1.80	0.321
Non-permanent AF	1.46	0.97–2.21	0.069	1.22	0.79–1.88	0.366
Vascular disease	0.98	0.69–1.39	0.886	0.86	0.60–1.23	0.398
Severity of stroke (NIHSS)|^a^	1.10	1.07–1.14	< 0.001	1.10	1.06–1.13	< 0.001
Smoking status	1.16	0.92–1.46	0.202	0.91	0.70–1.18	0.462

^a^ - National Institute of Health stroke scale

**Fig. 3:**
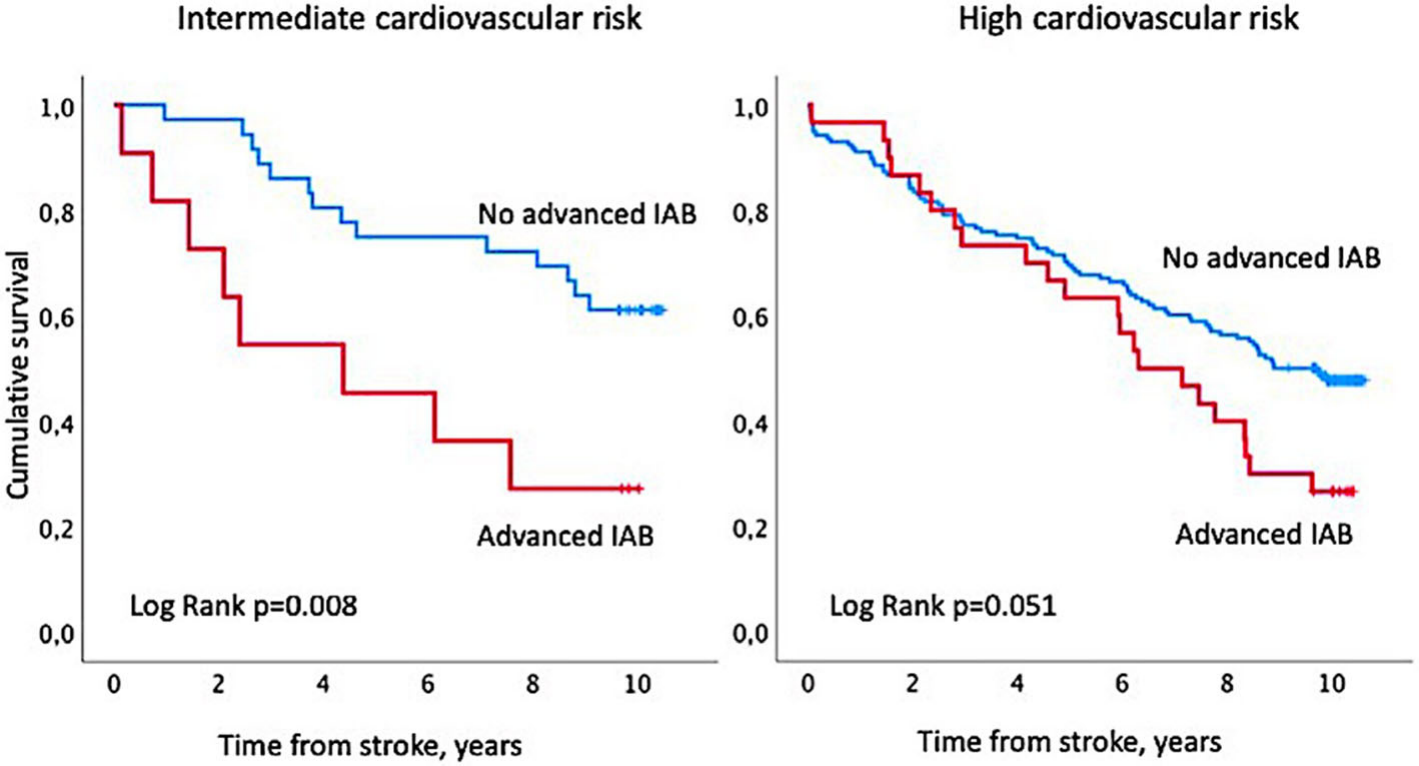
Kaplan-Meier survival curve indicating survival in patients with intermediate cardiovascular risk and in patients with high cardiovascular risk according to the presence and degree of interatrial block (IAB)

The Kaplan-Meier survival curves performed separately in patients with intermediate and high cardiovascular risk are presented in Fig. 3.

In patients with intermediate cardiovascular risk the difference in survival between patients with advanced IAB when compared to patients without advanced IAB was significant (log-rank *p* = 0.008), while in patients with high cardiovascular risk the difference in survival between patients with advanced IAB and patients without advanced IAB was not (log-rank *p* = 0.051).

When we compared cumulative survival in patients with advanced IAB and intermediate cardiovascular risk and in patients with advanced IAB and high cardiovascular risk using Kaplan-Meier method, we did not find any significant difference (log-rank *p* = 0.545).

In patients with intermediate cardiovascular risk the only independent predictors of all-cause mortality were advanced IAB and severity of stroke (Table 5), while in patients with high cardiovascular risk age at admission, male gender and severity of stroke, but not advanced IAB, independently predicted mortality.

**Table 5:** Multivariate Cox regression analysis showing the value of interatrial block (IAB) for prediction of all-cause mortality after first-ever ischemic stroke in patients with intermediate cardiovascular risk and high cardiovascular risk

Variable	Intermediate risk			High risk		
	HR	95% CI	P value	HR	95% CI	P value
Partial IAB vs no IAB	0.36	0.03–3.64	0.389	1.09	0.69–1.73	0.713
Advanced IAB vs no IAB	7.89	2.01–30.98	0.003	1.01	0.59–1.72	0.966
Age	1.02	0.97–1.06	0.494	1.11	1.08–1.14	< 0.001
Male gender	1.92	0.63–5.89	0.584	1.79	1.14–2.80	0.011
Cardiac failure	NA			1.31	0.64–2.68	0.458
Diabetes	NA			0.82	0.41–1.38	0.457
Hypertension	NA			1.24	0.75–2.06	0.405
Non-permanent AF	NA			1.26	0.80–2.00	0.321
Vascular disease	NA			0.86	0.56–1.31	0.484
Severity of stroke (NIHSS)|^a^	1.58	1.28–1.95	< 0.001	1.09	1.05–1.12	< 0.001
Smoking status	2.05	0.96–4.37	0.063	0.80	0.59–1.09	0.153

^a^ National Institute of Health stroke scale

### Oral anticoagulant therapy after ischemic stroke

High-risk patients were treated with OAC significantly more often than the intermediate-risk patients (*n* = 40, 21%, vs. *n* = 3, 6%; *p* = 0.019).

Treatment with OAC was not associated with differences in the long-term prognosis in the whole study sample (univariate HR for the probability of death 0.66 95% CI 0.41–1.09, *p* = 0.105), but was linked to a reduced probability of death in patients with history of non-permanent AF prior to stroke onset (*n* = 49, univariate HR for the probability of death in treated with OAC 0.22 95% CI 0.09–0.52, *p* = 0.001).

Among patients without history of non-permanent AF prior to stroke onset (*n* = 186) there were 75 patients with either partial IAB or advanced IAB (40%). Twenty two of these patients were treated with OAC: 9 patients with IAB (median time on OAC 3 years, IQR 0.5–9 years) vs. 13 patients without IAB (median time on OAC 7 years, IQR 1.5–9 years), *p* = 1.000.

**Fig. 4:**
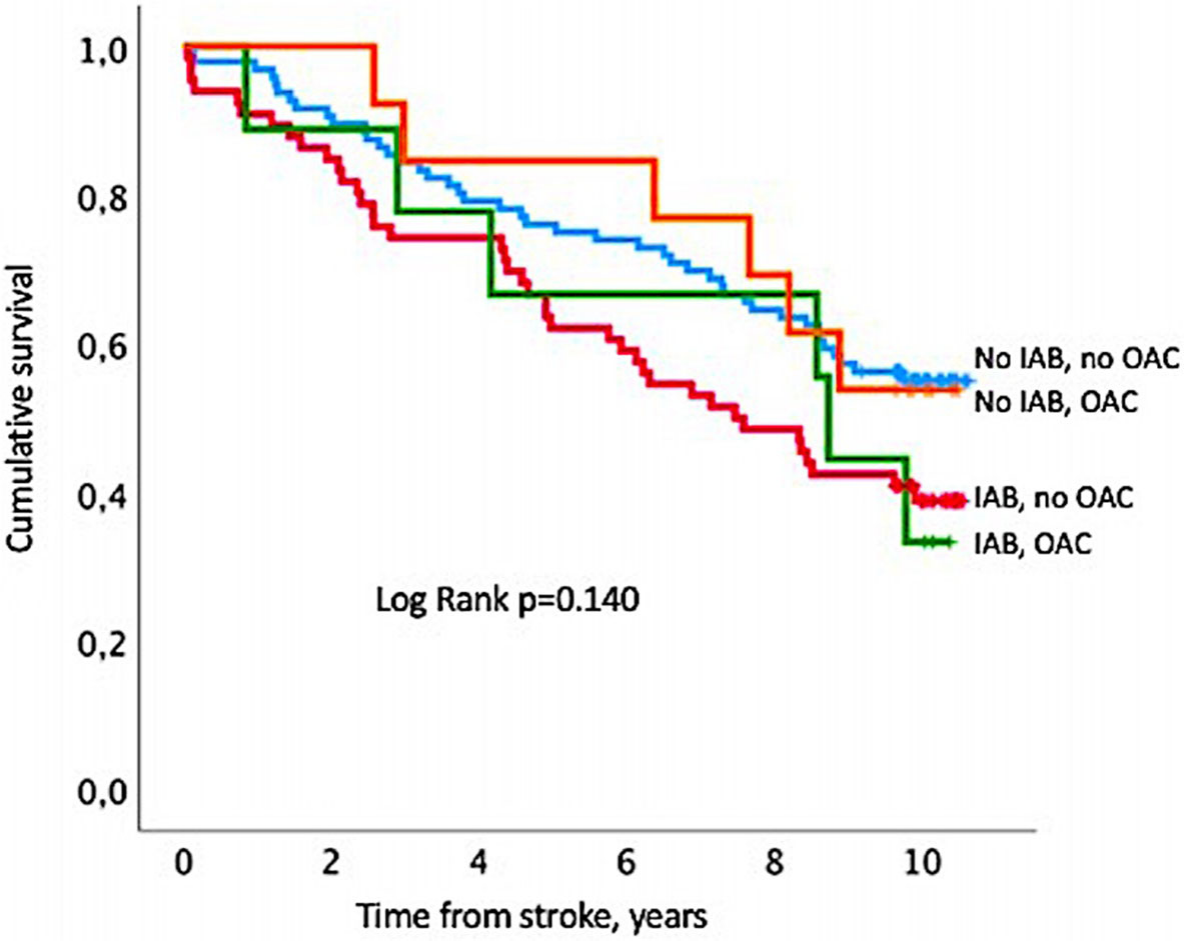
Kaplan-Meier survival curve indicating cumulative survival in atrial fibrillation-free patients with interatrial block (IAB) treated *(n = 9, green line)* and not treated *(n = 66, red line)* with oral anticoagulants (OAC) after stroke and without IAB treated *(n = 13, orange line)* and not treated *(n = 96, blue line)* with OAC, Log Rank *p* = 0.140

OAC therapy was not associated with significant differences regarding the long-term prognosis in IAB patients and patients with normal P-wave duration (Fig. 4). In the Kaplan-Meier curve analysis, cumulative survival in IAB patients on OAC was similar compared to IAB patients without OAC (log-rank *p* = 0.957) and did not significantly differ from that in patients without IAB with OAC (log-rank *p* = 0.413) and without OAC (log-rank *p* = 0.291). Among patients not treated with OAC, IAB was associated with a higher probability of death compared to those without IAB with the significance at the borderline level (log-rank *p* = 0.032).

### Cerebral infarction as a cause of death

Among 126 patients died during follow-up there were 26 (21%) patients whose cause of death was defined as cerebral infarction: 6 of 30 patients with advanced IAB (20%) vs 20 of 96 patients without advanced IAB (21%), *p* = 1.000. One of 6 patients with advanced IAB (17%) and 3 of 20 patients without advanced IAB (15%) were treated with OAC after discharge, *p* = 1.000.

## Discussion

We performed this post-hoc analysis on the high risk population of ischemic stroke survivors, who were prospectively enrolled in the observational Lund Stroke Registry study. We aimed to assess the prognostic value of IAB for prediction of all-cause mortality and evaluated the impact of OAC therapy on mortality in stroke patients with IAB.

We observed that the predictive value of advanced IAB depended on the clinical context. Though associated with a doubled mortality risk compared to patients without advanced IAB in an univariate analysis of the whole cohort, the effect was mostly confined to patients without comorbidities, in whom it was an independent predictor of the total mortality associated with eight-fold risk increase while in the high-risk population with concomitant comorbidities, it failed to demonstrate an independent predictive value. We also found that among stroke patients and IAB the administration of OAC was not associated with survival benefit.

### IAB as an electrocardiographic marker of underlying atrial fibrosis

IAB occurs when conduction between right and left atria slows down, however as long as propagation of electrical impulses through the Bachmann’s bundle persists, IAB is only manifested by prolongation of P-wave duration without significant alterations of P-wave polarity [2]. However, when structural abnormalities of atrial myocardium reach the degree when conduction over the Bachmann’s bundle is interrupted, the course of left atrial depolarization becomes reversed, which is reflected on surface ECG by the negative terminal deflection of the P-wave in the frontal plane, which has been defined as advanced IAB [1]. At admission with ischemic stroke, we observed IAB, i.e. P-wave duration > 120 ms, in *n* = 97 (41%) of patients, and advanced IAB in *n* = 41 (17%) of them. Our data was obtained using fully automated P-wave morphology assessment, which eliminated the need for interobserver variability assessment, and is in agreement with the literature, in which the prevalence of advanced IAB was reported to vary significantly from 0.5% in general population [3] and up to 26% in centenarians [12]. The latter study demonstrated age-related increased prevalence of advanced IAB. In agreement with that, our patients with IAB were older than those without IAB, and the oldest patient group were those with advanced IAB.

Increased prevalence of IAB with age has been explained by age-related increase in the degree of atrial fibrosis [13]. Atrial fibrosis may be manifested by its effect on atrial conduction, which can present as P-wave prolongation on ECG, including development of the advanced IAB pattern. Apart from age, other cardiovascular comorbidities, such as hypertension, heart failure and diabetes, have been associated with fibrosis development in earlier studies and visualized using delayed-enhancement magnetic resonance imaging [14]. Notably, in our study age was the only factor significantly associated with IAB, while none of cardiovascular comorbidities demonstrated a significant association with IAB.

### Advanced IAB and all-cause mortality

To the best of our knowledge the literature data concerning the association between advanced IAB and all-cause mortality are limited. In one population-based study the association of P-wave duration with the risk of cardiovascular and all-cause mortality was observed in a subgroup of subjects without cardiovascular comorbidities, i.e. low cardiovascular risk [5], which is supported by our findings of context-dependent performance of advanced IAB as an ECG-based risk indicator. In patients with advanced heart failure enrolled in the MADIT-II study, i.e. high cardiovascular risk, no association between P-wave duration and death from any cause was found [15].

The possible explanation of potential mechanisms for the relation between IAB and mortality refers to the data mostly showing the association of IAB with AF and ischemic stroke [5]. It is however plausible to also consider IAB as an ECG marker of atrial fibrosis that can be a result of age or caused by cardiovascular comorbidities and mediated through an increased atrial filling pressure, stretch, ischemia or inflammatory reaction [16]. In line with the earlier reported age-related fibrosis increase, in our study, IAB at the time of admission with ischemic stroke was associated only with age but not with cardiovascular comorbidities. Notably, Kaplan-Meier analysis in our study demonstrates similar cumulative survival in patients with advanced IAB regardless of comorbidity status, thus suggesting that those with advanced IAB without known comorbidities could have concealed structural abnormalities in the atria at the time of stroke, cardiovascular disorders and thus higher mortality risk. On the other hand, in patients with high cardiovascular risk and a high a priori risk of death, advanced cardiovascular comorbidity is likely to play a more prominent role for prognosis thereby making prognostic significance of IAB lower.

### Oral anticoagulant therapy

The benefit of OAC therapy in patients with high risk of thromboembolism without known AF is a topic of considerable contemporary scientific discussion that has led to initiation studies with novel OACs in patients with embolic stroke of undetermined source [17].

According to the atrial cardiomyopathy concept, atrial fibrosis per se may lead to increased risk of thromboembolism, and AF may according to this concept be considered a risk indicator rather than the cause of thromboembolism [18]. Therefore, even in the absence of documented AF, advanced cardiovascular risk profile may provide a rationale for administration of anticoagulant therapy. The presence of advanced IAB in patients with high risk of cardioembolic stroke may thus indicate a group of high risk who would benefit from OAC even without detected AF [19]. A reported association of advanced IAB with cardioembolic stroke [6] provides a rationale for this concept.

Contrary to those data we found that death rate due to cerebral infarction was similar in patients with advanced IAB and without advanced IAB indicating that advanced IAB was more likely a marker of high cardiovascular risk not being associated with increased embolic risk. Furthermore we did not find any benefit of OAC therapy in stroke patients with IAB and based on our findings could not advocate the hypothesis that advanced IAB might be useful in stratification of high risk patients for treatment with OAC. However, in our study OAC was expectedly associated with significantly reduced all-cause mortality among patients with AF, which supports the robustness of our data and ascertainment of study endpoints.

The absence of the association between the use of OAC therapy and all-cause mortality in our entire cohort is in agreement with the current opinion to the secondary prevention of ischemic stroke advocating the antiplatelet therapy unless an indication for OAC exists [20].

### Study limitations

The general limitation is that our study was retrospective and observational by design and not prespecified. ECG registration was not included in the protocol at inclusion in the study. However, the vast majority of patients had clinically motivated admission ECG stored in digital format, which was available for our analysis.

Our study is of limited size and therefore underpowered for detailed subgroup analysis of the relationship between the different degrees of interatrial block and long-term outcome. For this reason, patients with partial IAB were not included as a separate group in the Kaplan-Meier analysis.

Analysis with regard to IAB and OAC may result in insufficient power for drawing definite conclusions due to the low usage of OAC in our study sample. OAC were administered in a limited extent and not always in accordance with contemporary indications. However, it could be counterbalanced by the long follow-up time, which is not yet available for contemporary treated patients.

## Conclusion

In ischemic stroke survivors, advanced IAB was associated with all-cause mortality, with a eight-fold mortality risk increase in patients without additional cardiovascular comorbidities, but failed to demonstrate predictive value in patients with additional cardiovascular comorbidities.

## Abbreviations

AF: Atrial fibrillation
CI: Confidence interval
ECG: Electrocardiographic
HR: Hazard ratio
IAB: Interatrial block
IQR: Interquartile range 25–75%
LSR: Lund Stroke Register
NIHSS: National institute of health stroke scale
OAC: Oral anticoagulant
OR: Odds ratio
